# What Constitutes Authorship in the Social Sciences?

**DOI:** 10.3389/frma.2021.655350

**Published:** 2021-03-23

**Authors:** Gernot Pruschak

**Affiliations:** ^1^Department for Business Decisions and Analytics, University of Vienna, Vienna, Austria; ^2^Business School, Bern University of Applied Sciences, Bern, Switzerland

**Keywords:** academic incentive system, authorship, ethics in research, research tasks, specialization, survey research

## Abstract

Authorship represents a highly discussed topic in nowadays academia. The share of co-authored papers has increased substantially in recent years allowing scientists to specialize and focus on specific tasks. Arising from this, social scientific literature has especially discussed author orders and the distribution of publication and citation credits among co-authors in depth. Yet only a small fraction of the authorship literature has also addressed the actual underlying question of what actually constitutes authorship. To identify social scientists' motives for assigning authorship, we conduct an empirical study surveying researchers around the globe. We find that social scientists tend to distribute research tasks among (individual) research team members. Nevertheless, they generally adhere to the universally applicable Vancouver criteria when distributing authorship. More specifically, participation in every research task with the exceptions of data work as well as reviewing and remarking increases scholars' chances to receive authorship. Based on our results, we advise journal editors to introduce authorship guidelines that incorporate the Vancouver criteria as they seem applicable to the social sciences. We further call upon research institutions to emphasize data skills in hiring and promotion processes as publication counts might not always depict these characteristics.

## Introduction

The recent years brought large changes to academia. One aspect where we see this very clearly is the number of scientists working on and receiving credits for research projects. Whereas, from all papers published in the *American Economic Review* in 1950 only 8 percent inlcuded more than one author, 80 percent of all papers published in the *American Economic Review* in 2010 included more than one author (Hudson, 1996; Hamermesh, 2015). Having multiple authors working on the same research paper allowed scientists to divide the workload. Hence, Smith's (2007) concept of the division of labor also works in academia: Dividing research projects into individual tasks allows specialization. Scientists who are good in collecting and/or analyzing data might focus on these affairs while skilled writers might engage more in drafting the actual paper. It is therefore no wonder that the increase in co-authored papers also came along with a boost in academic productivity with more and more publications being published in less and less time (Lee and Bozeman, 2005; Engels et al., 2012; Fanelli and Lariviere, 2016). As publications and citations of publications represent the primary indicator of academic productivity because institutional funding often depends on them (Bloch and Schneider, 2016), multi-authored research projects required new measurement techniques for assessing researchers' productivity and impact (Carpenter et al., 2014). To cope with this issue, Cole and Cole (1973, p. 33) introduced a technique called “straight counts.” They only award publication and citation counts for the first author with all other authors not receiving those benefits (Cole and Cole, 1973). Similar to this approach, Shaw (1967) and Stallings and Singhai (1970) introduced techniques that give the first author a certain ratio of the counts and all other authors together the remaining ratio of the counts (e.g., for a paper with three authors the first author would receive half of the counts and the other two authors would receive each a quarter of the count). Yet these approaches relied on estimates as one could not know the actual degree to which each author engaged in the research process (Lindsey, 1980). In addition, these techniques did not seem reasonable for fields like Accounting or Economics in which authors are not ranked by contribution but alphabetically (Engers et al., 1999; Chan et al., 2009). To better assess researchers' impact and productivity, various editorials and articles have followed up on the discussions of author order and how to distribute publication and citation counts among co-authors (e.g., Floyd et al., 1994; Endersby, 1996; Macfarlane, 2017; Sauermann and Haeussler, 2017; Balkin et al., 2018). But while these are already highly contested topics, the basic underlying question of what actually constitutes authorship has not received much attention in the social sciences so far.

Previous studies have mainly focused on wrongdoings when assigning authorship (e.g., gift authorship and ghost authorship) highlighting the existence of such issues (Hamilton et al., 1997; Manton and English, 2006, 2008). Yet at least a part of these findings might derive from the fact that most of the studies applied authorship criteria not stemming directly from the social sciences to assess the individuals that wrongly received or did not receive authorship. As a case in point, Bartle et al. (2000) find that psychologists' perceptions of authorship criteria differ from universally valid criteria. For management research “existing guidelines and protocols from the sciences can aid in attributing authorship, but there are potential difficulties in extrapolating from the pure sciences to business given the variances in team size and the inherent tasks to be assigned” (Zutshi et al., 2012, p. 33).

This article therefore aims at unveiling the actual requirements social scientists set for authorship by conducting a worldwide empirical survey. The research question therefore is:

**Research question:** *In what research tasks do social scientists have to engage in order to receive authorship?*

The paper is structured as follows. The next section discusses authorship and potential research tasks leading to authorship. Based upon this literature background we stipulate our research hypotheses. We then explain the research method and the sample. Afterwards, we show the results of the statistical analysis. This is followed by the discussion of our findings. We conclude by outlining future research opportunities and calling upon academic societies, journal editors and research institutions to carefully address the definition of authorship.

## Literature Background and Research Hypotheses

### Definition of Authorship

“Before modern times, the authors (including scientific authors) of a work were regarded as both ‘originators’ and ‘authorities’ of that work.” (Claxton, 2005, p. 33) as they often were alone in the research and writing process. Yet, as shown above, research projects engaging multiple scientists have increased strongly over the recent years. As the academic incentive system focuses largely on publishing, the question of who gets to publish the results, in other terms who receives authorship, has become more and more important (Kendall and Campanario, 2016). The International Committee of Medical Journal Editors (ICMJE), also named the Vancouver group, took on this question in in 1985 (Leash, 1997). After long discussions the ICMJE introduced for the first-time authorship criteria that were applicable for multiple journals, societies and disciplines, the so-called Vancouver criteria (ICMJE, 1985 Smith, 1997). According to these criteria, “authorship credit should be based only on substantial contributions to (a) conception and design, or analysis and interpretation of data; and to (b) drafting the article or revising it critically for important intellectual content; and on (c) final approval of the version to be published” (ICMJE, 1988, p. 402). Only a person fulfilling all three requirements should receive authorship (Vuckovic-Dekic, 2003). Originally, predominantly medical journals applied the Vancouver criteria (Wager, 2007). However, the scientists publishing in those journals were not always aware of these criteria. As a case in point, Hoen et al. (1998) found that an overwhelming majority of the researchers publishing in the Dutch Journal of Medicine, a journal subscribing to the Vancouver criteria, were not aware of their existence. Interestingly, still about two thirds of the scholars fulfilled the Vancouver criteria despite not knowing them (Hoen et al., 1998). Using the Vancouver criteria as a starting point to identify authorship malpractices in the form of gift (authors not fulfilling the Vancouver criteria) and ghost (non-authors fulfilling the Vancouver criteria), several studies showed that a substantial number of published articles in various life-science disciplines violated those criteria (Flanagin et al., 1998; Mowatt et al., 2002; Wislar et al., 2011). Applying similar techniques to the social sciences, Pruschak and Hopp (2019) found that the author list of on average every third paper also violated the Vancouver criteria. As discussed above, this is neither surprising nor extremely problematic as the social sciences might possess and apply their own authorship criteria instead of the ones from the International Council of Medical Journal Editors.

Nevertheless, many large social scientific academic societies and publishers (e.g., the Academy of Management, Cambridge University Press, Elsevier, …) subscribe to the Committee on Publication Ethics (COPE) for guidance on academic practices^1^. According to the Guidelines for Publication Practice “there is no universally agreed definition of authorship” (COPE, 1999, p. 44). Yet the text continues by stating that “The award of authorship should balance intellectual contributions to the conception, design, analysis and writing of the study against the collection of data and other routine work. If there is no task that can reasonably be attributed to a particular individual, then that individual should not be credited with authorship” (COPE, 1999, p. 44). Assessing authorship disputes more specifically in the COPE Report 2003, COPE associate members Albert and Wager (2004) referred explicitly to the Vancouver criteria as the standard authorship criteria in life and social sciences. This goes in line with empirical evidence showing that life science and social science journal possess more often authorship guidelines than natural science journals with most of the existing authorship guidelines being in coherence with the Vancouver criteria (Resnik et al., 2016). Looking only at social science journals, all journals published by Wiley-Blackwell require authors to fulfill the Vancouver criteria (Bosnjak and Marusic, 2011). Hence, these criteria might also play a crucial role when assessing authorship in the social sciences. For example, the American Sociological Association (ASA) requires scholars to “take responsibility and credit, including authorship credit, only for work they have actually performed or to which they have made a substantial contribution” (ASA, 2018, p. 17). With the term “substantial contribution” appearing in both, the Vancouver criteria and in the criteria set by the ASA, this points toward the influence the ICMJE also possesses on the authorship definition in the social sciences.

Based on the importance of the three Vancouver criteria, we stipulate our first hypotheses that social scientist award authorship in accordance with the Vancouver criteria:

**Hypothesis 1a:** Social scientists receive authorship for participating in conception and design, or analysis and interpretation of data.

**Hypothesis 1b:** Social scientists receive authorship for drafting the article or revising it critically for important intellectual content.

**Hypothesis 1c:** Social scientists receive authorship if they give their final approval of the version to be published.

### Research Tasks and Authorship

Researchers nowadays more and more divide their labor and therefore often do not engage in every single research task (Engels et al., 2012). This requires a split of the Vancouver criteria into individual research tasks^2^. Hereby, we follow the structure of the archetypical academic research process (Miller and Salkind, 2002; Mowatt et al., 2002; Garrat and Li, 2005; Lancaster, 2005; Lee and Lings, 2008; Weathington et al., 2012). The tasks embedded in the research conception and design, or analysis and interpretation of data are the creation of the research design, the search for relevant literature, the analysis of the relevant literature, the data work including data preparation and data analysis as well as the description of the results (Mowatt et al., 2002; Garrat and Li, 2005; Bhattacherjee, 2012; Weathington et al., 2012). Writing the paper as well as reviewing and remarking the written paper represent the two tasks comprising the article drafting and article revising Vancouver criterion (Mowatt et al., 2002; Bhattacherjee, 2012; Zukauskas et al., 2018). Adding to this the approval of the final version completes the task list for the social scientific research publication process (Nolan and Rocco, 2009).

Creating the research design represents the first step when conducting research (Bhattacherjee, 2012). Hereby, scholars formulate a research question highlighting the phenomenon they want to investigate. They select the research approach and define the scope of the literature. In case of quantitative or qualitative research, scholars also set the ways and means of the data collection (Miller and Salkind, 2002). As the research design defines the sphere and shape of the research project, it is no wonder that scientists engaging in its creation often are the first authors, especially as they contribute to the whole research process from the early beginning until publication (Fine and Kurdek, 1993). Therefore, scholars creating the research design might receive authorship nearly every time. The following hypothesis refines this statement.

**Hypothesis 2a:** Social scientists receive authorship for creating the research design.

The research design closely relates to the literature search. Without knowing what research has already been conducted it is impossible to identify research gaps and formulate research questions (vom Brocke et al., 2009). In other words, “the literature review provides a starting point for research” (Seuring et al., 2005, p. 92). Hence, it is safe to say that reviewing/searching the literature represents an important task. Those scholars who carry this responsibility might therefore also receive authorship. Yet sometimes elderly scholars tend to outsource the search for literature to (research) assistants (Howe and Moses, 1999; Landrum and Nelsen, 2002; Turner, 2010). As authorship is occasionally incorrectly withheld from research assistants and doctoral students, this might negatively influence the relationship between scholars engaged in the search for literature and their authorship credits (Wislar et al., 2011). Overall, we still stipulate a positive connection between searching for literature and authorship, but the effect might be weaker than the effect of creating the research design.

**Hypothesis 2b:** Social scientists receive authorship for engaging in the search for literature.

After the literature base creation, a profound analysis of this base is necessary to identify appropriate theoretical frameworks because solid theory provides a stable foundation on which new concepts and research can build upon (Harriss, 1998; Lee and Lings, 2008; Onen, 2016). Furthermore, the widely-employed confirmatory research approach requires scholars to deduct hypotheses about their research outcomes from the existing literature before answering the research question empirically or experimentally (Lundberg, 1976; Jaeger and Halliday, 1998). As research hypotheses possess an enormous impact on the following research tasks due to them defining the concepts and relationships under investigation in detail, the literature analysis defines the ultimate shape of the research project (Weathington et al., 2012). This also explains why the the person(s) contributing most to a research project and frequently also the person(s) managing the communication flow between all authors often engage(s) in analyzing the literature (Kraut et al., 1987). Summing up, we might observe that scholars engaging in the literature analysis frequently receive authorship.

**Hypothesis 2c:** Social scientists receive authorship for engaging in the analysis of the literature.

The next task, the data work, is not relevant for theoretical or literary research projects as those two research approaches do not base upon data (Lancaster, 2005). In all other research projects, the data work represents the main step toward revealing the actual novel research results (Garrat and Li, 2005). Especially the data analysis represents often the most important aspect as the research findings and conclusions are the direct and indirect outcomes of this task (Mosteller and Tukey, 1977; Thonre, 2000). It is therefore no wonder that Hart (2000) finds that university librarians assess high importance to data analysis when assessing the distribution of authorship. Yet a study among education researchers and education research students shows that the latter ones are often willing to give authorship credits for data related tasks while faculty members are more reluctant (Welfare and Sackett, 2010). In addition, often students or research assistant conduct at least the data collection, especially in areas where this consumes a lot of efforts and time (e.g., interview-based research, manual survey distributions, …) (Marusic et al., 2011). As discussed previously, those are groups that are sometimes left out in the authorship distribution (Wislar et al., 2011). Combining both arguments of this paragraph, there might exist a small effect of being engaged in the data work on authorship.

**Hypothesis 2d:** Social scientists' engagement in the data work does weakly relate to whether they receive authorship.

Describing the results represents the last task of the first Vancouver criterium. This does not necessarily already have to take place as part of the paper writing. Quite the contrary, in multi-authored research projects scholars often note the first results and implications in catchword, feeds and bullet points which they then use to discuss them with their co-authors (Kallestinova, 2011; Wegener and Taggard, 2013). Hereby, the (first) description of the results substantially influences the discussion and hence also the research projects' implications and conclusion. Unfortunately, the current literature does not discuss the first descriptions of the results in more detail. This lack of information might derive from a lack of importance compared to the actual writing process (Hart, 2000). Nevertheless, we stipulate that describing the results might lead to authorship as the discussions of the first results often takes place between co-authors.

**Hypothesis 2e:** Social scientists receive authorship for describing the results.

The second Vancouver criterium refers to the actual writing and reviewing process. Writing the paper represents the first task. According to university librarians, this is the most important task in the whole research process (Hart, 2000). With the academic incentive system largely focusing on publishing and the competition for publication spots increasing, scholars have put more and more emphasis on the writing process (Lee and Lings, 2008; Weathington et al., 2012). The countless articles and books about academic writing aiming at helping scholars to improve their manuscripts further hint toward the importance of the actual writing process (e.g., Hall, 2008; Murray, 2013; Gustavii, 2017). Based on this evidence, writing the paper might be strongly related to receiving authorship.

**Hypothesis 2f:** Social scientists receive authorship for writing the paper.

Last, after finalizing the first draft (and possibly also after submitting the paper to journals for even a couple of times), co-authors, reviewers, contributors and/or other colleagues might remark the written paper (Elliott and Fischer, 1999). The quality and quantity of such remarks can differ tremendously. They range from copy-editing via comments on employed (statistical) methods up to critics on the theoretical framework or even the research design (Bedeian, 2004). Also, feedback from presentations held at conferences, workshops, etc. can count as remarks (Oester et al., 2017). Nevertheless, not all individuals giving remarks also count as authors (Strange, 2008). Frequently, researchers thank groups of people in the acknowledgments but do not mention everyone by their name (Yang, 2012). Remarks also usually come from peer-reviewers. Those also do not receive authorship as the authors do not know their identity (Moylan et al., 2014). Hence, simply giving remarks might not be sufficient enough to receive authorship. However, giving remarks and at the same time also engaging in the research project with another task might point toward authorship.

**Hypothesis 2g:** Social scientists receive authorship for remarking a paper if they are also engaged in at least one other research task.

## Research Method

To answer the research question and test the research hypotheses, we employed a large-scaled empirical survey among social scientists from various disciplines. We analyze the gathered data by applying logistic regressions to identify the tasks that induce the assignment of authorship.

### Survey Design and Distribution

We created the survey using the online tool Qualtrics. The questionnaire consisted of three parts. Out of these three parts, only the first two parts are relevant for this study. The first survey section contained demographic and academia related questions. For example, respondents should indicate their primary research discipline, their current job position as well as the geographical area in which their research institution is located. The second part of the questionnaire asked social scientists for the number of authors of and for the number of other persons contributing to their last published paper^3^. For each of these authors (up to a maximum of five authors) the respondents then received a task list and should checkmark those tasks in which the respective author actively participated. We chose five authors as the maximum to let participants answer the survey in a reasonable time frame. As a case in point, only 20% of the papers published in the American Economic Review in 2011 had more than four authors (Hamermesh, 2015). Nevertheless, we also asked respondents whose last published paper contained more than five authors to checkmark the tasks for the five authors who contributed most. Furthermore, the survey required respondents to also checkmark the task list for each non-author contributing actively to the paper (again up to a maximum of five contributors).

After creating and reviewing the questionnaire, we sent it to friendly colleagues asking for error-detection and feedback. We received only minor improvement suggestions and updated the survey correspondingly. Subsequently, we sent the survey to 275 participants of the 2018 European Accounting Association Annual Congress for pre-testing. The quantitative and qualitative responses did not point toward any irregularities and hence there was no need for changing the survey. This also explains why the results from the pre-testing are also included in the subsequent study.

In late summer 2018, we sent out the survey link to 63,240 individual email addresses of corresponding authors from papers published in discipline specific high impact journals and/or papers presented at large discipline specific societies between January 2010 and June 2018. These journals and societies lie within the fields of business, economics, information systems, law, operations research and statistics, political sciences, psychology and sociology^4^. After manually processing information from bounce-back emails, we sent out a reminder email 10 days later to an updated list of 50,342 scholars. We also manually processed bounce-back emails after the reminder wave and concluded that 48,015 valid email addresses of social scientists received the original survey and/or the reminder.

In total, we got 2,817 responses. This corresponded to a response rate of 5.87% which mirrored the response rates in previously conducted studies focusing on academic (mis-)conduct (Hopp and Hoover, 2017; Liao et al., 2018). Out of all respondents, 2,223 finished the questionnaire. However, as we asked for the authorship distribution in their last published paper, respondents with no published paper so far drop out of the sample. The same applies to observations containing at least one N/A answer in one of the variables employed in the analysis. Yet every respondent did not only assess their own tasks for their last published papers but the task for all (or up the top five) authors and contributors. This explains why our sample size lies above the number of full responses. More specifically, our analysis contains 6,131 authors and non-authors contributing to 1,728 papers^5^.

### Variables and Statistical Methods

*Author* represents our dependent variable. It is a dummy variable that is 1 if the respective individual received authorship and 0 otherwise. The first set of independent variables contain the individual research tasks: *Research Design* (1 if the respective individual engaged in creating the research design; 0 otherwise), *Literature Search* (1 if the respective individual engaged in the search for literature; 0 otherwise), *Literature Analysis* (1 if the respective individual engaged in the literature analysis; 0 otherwise), *Data Work* (1 if the respective individual engaged in data work; 0 otherwise), *Results Description* (1 if the respective individual engaged in describing the results; 0 otherwise), *Writing Paper* (1 if the respective individual engaged in the paper writing process; 0 otherwise), *Remarking Paper* (1 if the respective individual remarked the written paper; 0 otherwise) and *Approving Paper* (1 if the respective individual approved the final version of the paper to be published; 0 otherwise). We also condense these individual tasks into the three Vancouver criteria which represent the independent variables for the second part of the analysis. Hereby, *Conception and Design, or Analysis and Interpretation of Data* equals 1 if at least one of the following variables is also 1: *Research Design, Literature Search, Literature Analysis, Data Work* and/or *Results Description*. The Vancouver criterion *Drafting and/or Revising the Article* is assigned 1 if the variables *Writing Paper* and/or *Remarking Paper* equals 1. The Vancouver criterion *Final Approval* equals the variable *Approving Paper*.

As for the controls we apply mixed-effects models by grouping the observations into the individual published papers. These mixed-effects capture the individual paper-based differences (e.g., composition of research team, specific research institution) as well as similarities shared between groups of papers (e.g., research field, geographical region, size of research team) (Gelman and Hill, 2006). More specifically, we employ the melogit command of Stata 16 to assess the relationship between fulfilling the Vancouver criteria and engaging in the research tasks on the one hand and the awarding of authorship on the other hand.

## Results

### Descriptive Statistics

Table 1 shows the descriptive statistics for the Vancouver criteria^6^. Out of the 6,131 individuals, 4,795 are authors and 1,336 are non-authors contributing to 1,728 papers. While more than 90% of the authors fulfill the *Conception and Design, or Analysis and Interpretation of Data* criterion, this only applies to a minority of the non-authors. These ratios shift only slightly when we consider *Drafting and/or Revising the Article*. Still nearly 90% of the authors engage in this criterion. At the same time, slightly less than one third of the non-authors draft and/or revise the article. The responses for the *Final Approval* differ from the general theme of the first two criteria with only about three quarters of the authors and only ten percent of the non-authors giving final permission for the article submission.

**Table 1:** Number of authors and fulfillment of vancouver criteria.

	**Author**		
	0	1	Total
Conception and design, or analysis and interpretation of data			
0	739	381	1,120
1	597	4,414	5,011
	1,336	4,795	6,131
Drafting and/or revising the article			
0	944	573	1,517
1	392	4,222	4,614
	1,336	4,795	6,131
Final approval			
0	1,198	1,038	2,236
1	138	3,757	3,895
	1,336	4,795	6,131

After elaborating on the fulfillment of the Vancouver criteria, Table 2 splits up those criteria into individual research tasks. We find that not all authors engage in all tasks but instead they tend to focus on certain tasks. *Approving Paper* is the task with the highest share of authors with more than 75% of them giving their consent for submission. In turn, only slightly <60% of the authors engage in the *Data Work*. At the same time, this is the task with the highest participation rate among non-authors with one third of them contributing to the *Data Work*. Unsurprisingly, non-authors take only rarely part in the *Paper Writing*. The same also applies to *Results Description* and *Approving Paper*.

**Table 2:** Number of authors and engagement in research tasks.

	**Author**		
	0	1	Total
Research design			
0	1,180	1,572	2,752
1	156	3,223	3,379
	1,336	4,795	6,131
Literature search			
0	1,145	1,741	2,886
1	191	3,054	3,245
	1,336	4,795	6,131
Literature analysis			
0	1,186	1,687	2,873
1	150	3,108	3,258
	1,336	4,795	6,131
Data work			
0	896	1,988	2,884
1	440	2,807	3,247
	1,336	4,795	6,131
Results description			
0	1,209	1,585	2,794
1	127	3,210	3,337
	1,336	4,795	6,131
Writing paper			
0	1,218	1,307	2,525
1	118	3,488	3,606
	1,336	4,795	6,131
Remarking paper			
0	988	1,132	2,120
1	348	3,663	4,011
	1,336	4,795	6,131
Approving paper			
0	1,198	1,038	2,236
1	138	3,757	3,895
	1,336	4,795	6,131

Tables 3, 4 contain the pairwise correlation coefficients of all relevant variables. Both tables indicate that authorship as well as the fulfillment of the Vancouver criteria or the engagement in different research tasks relate to each other positively with all correlation coefficients being statistically significant. Yet our analysis does not seem to suffer from multicollinearity with the highest correlation coefficient in Table 3 corresponding to 0.6155 and the highest correlation coefficient in Table 4 corresponding to 0.7508. These values are below the problematic cases of 0.8–0.9 (Mansfield and Helms, 1982). Interestingly, while most of the research tasks associated coefficients in Table 4 range between 0.4 and 0.6, the coefficients of *Data Work* are the smallest ones among all variables with the pairwise correlation between *Data Work* and *Remarking Paper* amounting only to 0.0830 as the extreme case.

**Table 3:** Correlation matrix of vancouver criteria.

	**Author**	**Conception and design, or analysis and interpretation of data**	**Drafting and/or revising the article**	**Final approval**
Author	1.0000			
Conception and design, or analysis and interpretation of data	0.5061	1.0000		
Drafting and/or revising the article	0.5617	0.4225	1.0000	
Final approval	0.5834	0.4319	0.6155	1.0000

*Pairwise correlations based on 6,131 observations. All correlations are statistically significant at p < 0.001*.

**Table 4:** Correlation matrix of research tasks.

	**Author**	**Research design**	**Literature search**	**Literature analysis**	**Data collection and prepar-ation**	**Results descrip-tion**	**Writing paper**	**Remarking paper**	**Approving paper**
Author	1.0000								
Research design	0.4610	1.0000							
Literature search	0.4085	0.4406	1.0000						
Literature analysis	0.4433	0.4655	0.7508	1.0000					
Data work	0.2118	0.3006	0.3158	0.3095	1.0000				
Results description	0.4761	0.5049	0.4920	0.5439	0.3607	1.0000			
Writing paper	0.5361	0.5308	0.5633	0.6015	0.2764	0.6224	1.0000		
Remarking paper	0.4370	0.4022	0.3236	0.3598	0.0830	0.3834	0.4487	1.0000	
Approving paper	0.5834	0.5112	0.3975	0.4503	0.1780	0.4919	0.5440	0.6090	1.0000

*Pairwise correlations based on 6,131 observations. All correlations are statistically significant at p < 0.001*.

### Mixed-Effects Analysis

To assess the research hypotheses, Table 5 shows the results from a mixed-effects logistic regression with *Author* as the dependent variable and the Vancouver criteria as the explanatory variables. Model 1, Model 2 and Model 3 each contain one criterion and the respective log odds are positive and statistically significant in all three models. Model 4 combines all three independent variables into one regression. We show that fulfilling the *Conception and Design, or Analysis and Interpretation of Data*, the *Drafting and/or Revising the Article* as well as the *Final Approval* increases the likeliness of receiving authorship. Hence, we find support for Hypotheses 1a, 1b, and 1c. We also compare the log odds in Model 4 using z-tests with two independent samples to assess the relative strength of the effects of the Vancouver criteria. The results indicate that the effects of fulfilling the *Conception and Design, or Analysis and Interpretation of Data* and the *Drafting and/or Revising the Article* do not differ from each other (Chi2 = 0.88; *p* = 0.3490). However, the effect of giving *Final Approval* is statistically significantly stronger than the effect of fulfilling the *Conception and Design, or Analysis and Interpretation of Data* (Chi2 = 12.86; *p* = 0.0003) and *Drafting and/or Revising the Article* (Chi2 = 16.02; *p* = 0.0001).

**Table 5:** Effects of vancouver criteria on authorship.

	**Model 1**	**Model 2**	**Model 3**	**Model 4**
	**Author**	**Author**	**Author**	**Author**
Conception and design, or analysis and interpretation of data	3.770^***^			2.460^***^
	(0.140)			(0.164)
Drafting and/or revising the article		4.459^***^		2.252^***^
		(0.157)		(0.162)
Final approval			5.037^***^	3.300^***^
			(0.181)	(0.180)
Chi2	725.77	808.76	774.52	782.91
*p > * Chi2	0.000	0.000	0.000	0.000
Observations	6,131	6,131	6,131	6,131

*** *p < 0.001*.

*Coefficients correspond to the marginal effects for the independent variables calculated at the mean levels of the remaining variables derived from mixed-effects logistic regressions with standard errors in parentheses*.

The models in Table 6 also derive from mixed-effects logistic regressions with *Authors* as the dependent variable. Yet these models possess the individual research tasks as explanatory variables. Model 5, Model 6, Model 7, Model 8 and Model 9, Model 11 and Model 12 as well as Model 14 each again only contain one independent variable. They show that engaging in every research task might increase the likeliness of receiving authorship. When we consider Model 10 (including all research tasks included in the *Conception and Design, or Analysis and Interpretation of Data* criterion) and Model 13 (including both, *Drafting and/or Revising the Article*), we find that all research tasks except for *Data Work* remain statistically significant. Model 15 incorporates all research tasks. Hereby, we again find statistically significant positive effects for all research tasks except for *Data Work*. This confirms Hypotheses 2a, 2b, 2c, 2e, and 2f. Notably, the effect is slightly weaker for engaging in the *Literature Search* and/or in the *Literature Analysis* than for the other tasks (*p* < 0.01 instead of *p* < 0.001). To assess Hypothesis 2f stipulating that *Remarking Paper* only induces authorship if it comes along engagement in at least another research task, Model 16 includes the interaction term *Remarking Paper and other research task(s)* (1 if individual gave remarks and also engaged in at least one other research task; 0 otherwise) as an independent variable. Whereas, this model contains a significant negative effect of *Remarking Paper* on receiving authorship, the effect of the interaction term on authorship is highly significantly positive. We therefore find support for Hypothesis 2f.

**Table 6:** Effects of research tasks on authorship.

	**Model 5**	**Model 6**	**Model 7**	**Model 8**	**Model 9**	**Model 10**	**Model 11**	**Model 12**	**Model 13**	**Model 14**	**Model 15**	**Model 16**
	**Author**	**Author**	**Author**	**Author**	**Author**	**Author**	**Author**	**Author**	**Author**	**Author**	**Author**	**Author**
Research design	3.546^***^					2.148^***^					0.942^***^	0.854^***^
	(0.131)					(0.143)					(0.161)	(0.162)
Literature search		3.129^***^				0.972^***^					0.552^**^	0.485^*^
		(0.123)				(0.168)					(0.191)	(0.191)
Literature analysis			3.440^***^			1.271^***^					0.616^**^	0.600^**^
			(0.130)			(0.173)					(0.200)	(0.201)
Data work				1.271^***^		−0.096					0.264	0.159
				(0.085)		(0.123)					(0.138)	(0.139)
Results description					3.934^***^	2.358^***^					1.106^***^	1.058^***^
					(0.148)	(0.158)					(0.178)	(0.179)
Writing paper							4.333^***^		3.784^***^		1.539^***^	1.458^***^
							(0.153)		(0.158)		(0.181)	(0.183)
Remarking paper								3.243^***^	2.344^***^		0.734^***^	−0.676^*^
								(0.122)	(0.137)		(0.157)	(0.294)
Approving paper										5.037^***^	2.766^***^	2.382^***^
										(0.181)	(0.186)	(0.193)
Remarking paper and other research task(s)												1.930^***^
												(0.335)
Chi2	736.31	645.54	699.61	225.26	707.35	759.11	798.56	705.23	782.50	774.52	786.90	791.17
*p > * Chi2	0.000	0.000	0.000	0.000	0.000	0.000	0.000	0.000	0.000	0.000	0.000	0.000
Observations	6,131	6,131	6,131	6,131	6,131	6,131	6,131	6,131	6,131	6,131	6,131	6,131

* *p < 0.05*;

** p < 0.01

*** *p < 0.001*.

*Coefficients correspond to the marginal effects for the independent variables calculated at the mean levels of the remaining variables derived from mixed-effects logistic regressions with standard errors in parentheses*.

Table 7 allows a more detailed comparison of the importance of participating in specific research tasks for receiving authorship by applying z-tests for two independent samples on all pairwise compositions of the coefficients derived from Model 15 in Table 6. *Approving Paper* possesses significantly the strongest positive effect on receiving authorship out of all research tasks. Besides this, the task *Writing Paper* also relates strongly to authorship with only the effect of engaging in the *Research Design* not being significantly weaker than the effect of *Writing Paper*. Interestingly, the effects of *Literature Search* and *Literature Analysis* do not differ significantly from the effects of *Research Design* and *Remarking Paper* albeit their higher *p-*values in Model 15 in Table 6. The effect of *Results Description* also does not differ significantly from the effect of *Literature Analysis*, but it is significantly stronger than the effect of *Literature Search*.

**Table 7:** Comparison of strength of the marginal effects from model 15 in Table 6.

	**Research design**	**Literature search**	**Literature analysis**	**Data collection and preparation**	**Results description**	**Writing paper**	**Remarking paper**	**Approving paper**
Research design	–							
	–							
Literature search	2.36	–						
	0.1244	–						
Literature analysis	1.54	0.03	–					
	0.2146	0.8520	–					
Data work	9.33	1.44	2.02	–				
	0.0023	0.2300	0.1552	–				
Results description	0.43	4.41	1.54	12.12	–			
	0.5143	0.0358	0.2146	0.0005	–			
Writing paper	5.38	12.65	10.28	30.94	2.37	–		
	0.0203	0.0004	0.0013	0.0000	0.1233	–		
Remarking paper	0.81	0.55	0.21	5.96	2.42	10.88	–	
	0.3693	0.4598	0.6434	0.0146	0.1197	0.0010	–	
Approving paper	49.29	71.69	61.84	120.25	40.74	22.16	53.02	–
	0.0000	0.0000	0.0000	0.0000	0.0000	0.0000	0.000	–

*First rows correspond to the Chi^2^-values for a z-test with two independent samples on the respective marginal effects from Model 15 in Table 6*.

*Second rows show the corresponding p-values*.

### Robustness Checks

We conduct a variety of robustness tests. First, we run all mixed-effects model also as fixed-effects models to account for the fact that every single research paper constitutes its own individual piece of work (Bachrach et al., 1998). This only leads to a single parameter change: When including the interaction term in Model 16 the negative effect of *Remarking Paper* on authorship is not significant anymore. Second, we run all models excluding the task *Data Work* to also include theoretical researchers in our analysis (*N* = 6,281). Yet the remaining effects stay within the same direction and significance levels as in our primary analysis. Third, we additionally exclude *Results Description* to also cover literate analyses (*N* = 6,374). Again, our findings for the remaining tasks persist. Fourth, running all models with robust standard errors does not change any significance levels. Fifth, running standard OLS regressions without mixed-effects returns the same implications. Last, the variance inflation factors derived from the OLS models lie below 3 for all variables except for the interaction term. Hence, our analysis does not suffer from multicollinearity as this is below the “conservative threshold of 5” (Alauddin and Nghiemb, 2010, p. 351).

## Discussion

### Summary of Results

We find that the fulfillment of each of the Vancouver criteria possesses a positive effect on receiving authorship. Whereas, the increase in the likeliness of receiving authorship is the same for researchers participating in the conception and design or analysis and interpretation of data compared to researchers drafting or revising the article, scholars approving the final paper version before submission have higher chances of receiving authorship. This result is little surprising for several reasons. First, approving the paper for submission represents a single task while the other two Vancouver criteria consist of multiple tasks. This reduces by definition the chances of non-authors to fulfill this criterion as we would obviously expect non-authors to engage in less tasks than authors. Therefore, non-authors might more often fulfill criterions where they only need to participate in one out of several tasks than in a single specific task. Second, researchers who have a say in whether a paper shall be submitted might often represent core members of the research team. Third, changes in authorship are usually more difficult after paper submission (Tarkang et al., 2017). Hence, those scholars who are authors at that point in time, usually are also authors in the published version of the paper. Last, researchers disagreeing with the paper submission, for example because they perceive the findings to be or controversial or not robust, might drop out of the author list by not participating in this stage instead of blocking the whole publication process.

Assessing the effects of the research tasks on authorship assignments in the social sciences, the results highlight that participation in all listed research tasks except for data work increase the likeliness of receiving authorship. This corresponds to the results in Bartle et al. (2000) who found that psychologists perceive that participating in the data collection does not constitute a pre-requisite for receiving authorship. We add to their results by showing that substantially more non-authors engage in the data work than in other research tasks in the social sciences. This might point toward the phenomenon that a substantial share of the individuals engaging in the data work do not participate in any other research task. In short, our findings suggest that social scientists regard mere data work contributions as not enough for authorship. This opinion fits perfectly the Vancouver criteria. Yet in many experimental and empirical research projects the data collection, data preparation and data analysis require high efforts and lots of time (Garrat and Li, 2005; Lancaster, 2005). Hence, articles should at least mention the contributors engaging in the data work explicitly in the acknowledgments.

In line with our results from the Vancouver criteria, approving the paper represents the most important task for receiving authorship. The second most important task is writing the paper. This again corresponds to the previously assessed opinions of psychologists (Bartle et al., 2000) and might derive directly from the academic incentive system that focuses largely on publishing (McGrail et al., 2006). Researchers nowadays can have the best research ideas and the best datasets, yet they will still have a hard time surviving in academia if they do not possess the skills to take those ideas and datasets and put them into a publishable format (Murray, 2013). Of course, one could now argue that research articles and book chapters represent not the only publishing format in academia. As a case in point, data publishing has gained more and more attention in the recent years (Kidwell et al., 2016). However, more than 85% of shared datasets remain uncited (Peters et al., 2016). As appointment procedures for faculty positions as well as tenure criteria often depend upon publications and citation counts, publishing articles still represents a critical pillar for academic success (McGrail et al., 2006). This explains why the scholars writing the paper also very often receive authorship.

The tasks describing the results and creating the research design possess the third strongest effects on authorship assignments. On the one hand, the importance of the creation of the research design is unquestionable. This task represents a very crucial step for any research project as it starts, defines, outlines and streamlines the scientific process (Fine and Kurdek, 1993; Miller and Salkind, 2002; Bhattacherjee, 2012). On the other hand, the importance of describing the results might connect to the importance of paper writing as the results description might include the drafting of the results section. Furthermore, describing the results might also comprise the creation of figures and tables, core elements of nearly all experimental and empirical research articles. Hence, describing the results represents a critical task in the paper creation process, especially in the light of social scientific journal editors preferring to publish novel and significant findings (Ioannidis, 2005).

Scholars searching for literature as well as analyzing the literature also possess increased odds for receiving authorship. Yet the effects on authorship are the weakest among all related tasks. The fact that sometimes elderly scholars tend to outsource literature tasks to assistants might provide an explanation for this phenomenon (Landrum and Nelsen, 2002; Turner, 2010; Howe and Moses 1999). Furthermore, splitting up the literature search and literature analysis among multiple researches is not reasonable in some research projects. To build a comprehensive and detailed knowledge base, scholars are required to read and process the full relevant list of existing research (vom Brocke et al., 2009). Hence, assigning the literature analysis to a specific person seems reasonable because this keeps efficiency levels high by enabling specialization. The assigned individual, however, might only engage in the literature search and/or literature analysis. As other scholars might contribute to more task of the research projects, she or he might not receive authorship but instead credits in the acknowledgments.

The basic analysis also indicates that remarking a paper increases the chances of receiving authorship. However, when we explicitly consider the combined contribution of remarking the paper and engaging in at least another research task, we show that reviewing the paper does not generally induce higher chances of receiving authorship. Instead, only researchers already engaging in at least one other research task increase their odds to receive authorship by also remarking the paper. This seems reasonable because besides co-authors also often colleagues, conference participants and peer-reviewers give feedback and remarks (Yang, 2012; Moylan et al., 2014; Oester et al., 2017). Yet we show that on average social scientists consider such remarks as not enough to award authorship to those giving them.

### Impact on Academia

This study provides a broad range of implications for various stakeholders in academia. We show that the Vancouver criteria are applicable to the social sciences and that many social scientists already award authorship in accordance to them. Considering that journals published by Wiley-Blackwell already apply the Vancouver criteria, we call upon other journal publishers, journal editors as well as academic societies to also base their authorship guidelines upon these criteria (Bosnjak and Marusic, 2011). In turn, journals and academic societies need to train reviewers and editors to detect breaches of those guidelines. To ease the detection of authorship malpractices, we suggest that journals and academic societies should introduce standardized author contribution disclosure tools like CRediT (Allen et al., 2019), especially as our results show that standardized task lists explain authorship assignments also in the social sciences. Introducing such tools would allow reviewers, editors, and publishers to assess authorship eligibility directly for each contributor to a research project. Furthermore, we believe that journals should require each author to approve the submission of a paper separately as social scientists assign high importance to this task.

Our study finds that social scientists try to profit from specialization by dividing their labor. Instead of multi-authored teams where everyone works on every task, we point out that research teams tend to distribute certain tasks to each person. The distributed tasks do not necessarily overlap implying that it might happen that a single researcher is in charge of a certain task although all authors together share the credits and responsibilities coming along with authorship. In the infamous example of Diederik Stapel, a psychologist who conducted major data fraud, his co-authors relied on the (nearly perfect) (experimental) data and did not supervise the experiments themselves (Zwart, 2017). “If Stapel was solely to blame for making stuff up, the report stated, his peers, journal editors and reviewers of the field's top journals were to blame for letting him get away with it” (Bhattacharjee, 2013). In the setting of multi-authored papers, we therefore call upon social scientists to not blindly trust co-authors in their tasks but instead oversee each other to detect errors and fraudulent behavior. If possible, social scientists should apply the four-eyes principle for each research task.

Last, we call upon research institutions and other employees of social scientists to shift away from solely counting publications and citations when assessing job applications and career advancements. We find that conducting data work does not lead to authorship. Yet the data work represents an important task in qualitative and quantitative research that also requires skills and training (Mosteller and Tukey, 1977; Thonre, 2000). With the demand for data scientists increasing strongly in the private sector, universities need to offer data focused faculty staff members attractive alternatives even if they possess not the best writing skills as they still enhance research overall if they work together in teams where others conduct the writing part (Davenport and Patill, 2012).

### Limitations and Future Research

No study is perfect. This also applies for this study although we included a variety of robustness and validity checks. First, our results derive from paper-based data. We asked scholars for information about every author and contributor (up to five each) of their last published paper. We limited the questionnaire to the five most important authors and contributors to avoid respondents to drop out because the survey would require too much time and effort. Consequently, we cannot infer to authorship compositions and task divisions for large-scaled research projects. While large collaborations with more than five authors still occur only rarely in the social sciences, future research could investigate whether authorship distributions differ in large teams compared to smaller ones. Moreover, we did not employ detailed demographic questions (e.g., age, faculty position, cultural background, …) for every (non-)author. The inclusion of such questions would have lengthened the questionnaire considerably, leading to more scholars quitting the survey. In addition, not every scholar might be aware of the detailed demographic and/or job-related characteristics of all other authors and contributors. In turn, asking for these characteristics could result in more respondents choosing the N/A option which would shrink the sample. Nevertheless, the application of mixed-effects regression controls for the different composition of research teams on the one hand (fixed-effects) but also incorporates the fact that groups of papers also share similarities like research fields or journal styles (random-effects).

Second, our data bases upon self-reported task participation and authorship distribution. The questionnaire did not ask respondents to indicate the paper they were referring to in an effort to keep the survey anonymous. Yet as authorship represents an important but at the same time also contested topic among social scientists, our data might contain socially desirable responses (Braun and Stallworth, 2009). This response bias is a common problem when conducting research in the area of academic (mis)conduct and questionable research practices (Spaulding, 2009).

Third, the study might suffer from sample selection as respondents voluntarily answered a survey they received via emails. Hence, social scientists who are interested in authorship distribution and similar topics might be more likely to answer the survey than social scientists with other interests. However, scholars, who, for example, are interested in authorship topics might more often be aware of the Vancouver criteria and in turn might more often award authorship in accordance to them. Thus, our findings provide only an upper boundary on the applicability of the Vancouver criteria to the Social Sciences.

Last, our analysis does not provide valid proof for direct causal effects. In fact, it might happen that endogeneity in the form of observable and unobservable omitted selection might bias our results. This would occur if there exists at least one factor that correlates with both, the dependent variable, receiving authorship, and the independent variables, the Vancouver criteria and/or the research tasks (Antonakis et al., 2010). For instance, research assistants might more often engage in the data collection and at the same time receive authorship less often (Marusic et al., 2011; Wislar et al., 2011). This selection effect might bias the *Data Work* marginal effects. Future studies could collect more data on authors and contributors and employ advanced statistical techniques like propensity score matching to assess the causality of our effects.

Besides collecting more data on participants, it would also be interesting to directly contact all listed authors and contributors mentioned in the acknowledgments asking them for the tasks they engaged in during the paper creation. This would allow for a comparison of whether the assessments undertaken by one author in this study depict the actual task distributions. Moreover, it would be interesting to move beyond authorship and focus on contributors. Hereby, a fruitful research question could be what differentiates contributors mentioned in the acknowledgments from those not mentioned in the acknowledgments.

On a more general note, upcoming research could also reduce socially desirable responding by employing item-sum-techniques that increase respondents perceived anonymity (Trappmann et al., 2014). Topicwise, future studies should investigate why and how scholars divide their labor. Does more pressure stemming from fiercer competition and an academic incentive system focusing more and more on publishing provide the only explanation or do other factors like new technologies, the vanishing of language barriers, … also influence social scientists' degree of specialization (Cox and Pinfield, 2014; Di Bitteti and Ferreras, 2017). In this context, one could also look at whether the same scholars always engage in the same research tasks or whether scholars take on different research tasks in different research projects.

Last, future research could go beyond investigating authorship by specifically looking at contributors. Hereby, one could ask for the requirements contributors must meet in order to be mentioned in the acknowledgments. Based on this it would be interesting to see whether acknowledgments give contributors (small) advantages when assessing academic careers.

## Conclusion

This research project was triggered by the stark contrast between the relatively high amount of social scientific literature discussing authorship order as well as the implications of receiving authorship and the relatively few social scientific literary pieces available dealing with the definition and assignments of authorship. Surveying social scientists, we show that the Vancouver criteria seem applicable as a valid general standard also to the social sciences. In addition, we find that social scientists benefit from specialization by dividing research projects into research tasks and distributing them among (individual) research team members. We highlight that data work as well as reviewing and remarking papers do not seem to influence whether social scientists receive authorship. Consequently, we advise research institutions to emphasize data skills in hiring and promotion processes. In addition, we call upon journal publishers and journal editors to introduce authorship guidelines that preferably incorporate the Vancouver criteria. Last, we put scholars themselves in charge of crosschecking the work done by their co-authors to detect errors and fraudulent behavior.

## Data Availability Statement

The data that support the findings and conclusion of this paper are available from the corresponding author upon request. The dataset is not made publicly available to ensure respondents' anonymity.

## Ethics Statement

Ethical review and approval was not required for the study on human participants in accordance with the local legislation and institutional requirements. Written informed consent for participation was not required for this study in accordance with the national legislation and the institutional requirements.

## Author Contributions

GP was involved in the data collection as well as manuscript writing and revising.

## Conflict of Interest

The author declares that the research was conducted in the absence of any commercial or financial relationships that could be construed as a potential conflict of interest.

## Footnotes

^1^A list of the subscribers is available online at: https://publicationethics.org/members.

^2^We only split up the first two Vancouver criteria as the approval of the paper submission/publishing represents already a single task.

^3^The questionnaire did not ask respondents to indicate the title/journal of paper they were referring to in an effort to keep the survey anonymous.

^4^A full list of all journals and societies is available upon request from the corresponding author.

^5^To preserve respondents' anonymity, we were not able to track who responded to the survey and who did not respond. Therefore, we were not able to conduct a non-response analysis. We address this issue by comparing early to late respondents. Supplementary Table 10 in the Appendix shows the full models for early and late respondents. This analysis reveals that early respondents indicated more significantly more often than late respondents that authors engaged in the data work.

^6^We do not include researchers' demographic as the questionnaire did not ask respondents to indicate those characteristics for every co-author and contributor. The inclusion of such questions would have lengthened the questionnaire considerably, leading to more scholars quitting the survey. In addition, not every scholar might be aware of the detailed demographic and/or job-related characteristics of all other authors and contributors. Nevertheless, Supplementary Tables 8, 9 in the Appendix show the demographic characteristics of the direct respondents (the corresponding authors).
