# Supplementary material for: What Constitutes Authorship in the Social Sciences?
Source: Front Res Metr Anal. 2021 Mar 23;6:655350. doi: 10.3389/frma.2021.655350 (PMC8028407; doi:10.3389/frma.2021.655350)
Supplement: Supplementary file 1 [file Data_Sheet_1.docx]

**APPENDIX**

**TABLE 8
Descriptive Statistics for Dichotomous Variables for Corresponding Authors**

| Total Respondents: | 1,728 | 100,00% |
| --- | --- | --- |
| Female | 569 | 32.93% |
| Anglophone (British Isles, North America, Australia & NZ) | 656 | 37.96% |
| Continental Europe | 705 | 40.80% |
| Developing Countries (Latin America, Africa, Southeast Asia) | 249 | 14.41% |
| PhD Students | 129 | 7.47% |
| Professors | 932 | 53.94% |
| Editors | 444 | 25.69% |
| Business Researchers | 599 | 34.66% |
| Economics and Finance Researchers | 233 | 13.48% |
| Computer, Operations and Statistics Researcher | 269 | 15.57% |
| Political Scientists | 173 | 10.01% |
| Psychologists | 105 | 6.08% |
| Sociologists | 142 | 8.22% |

**TABLE 9
Descriptive Statistics for Interval-Scaled Variables for Corresponding Authors**

|  | N | Mean | Standard Deviation | Minimum | Maximum |
| --- | --- | --- | --- | --- | --- |
| Age | 1,728 | 46.57 | 12.64 | 21 | 93 |
| Academic Working Years | 1,728 | 17.48 | 12.23 | 0 | 63 |
| Papers Published | 1,728 | 6.87 | 7.82 | 1 | 60 |
| Reviews Written | 1,728 | 7.26 | 9.56 | 0 | 60 |

**TABLE 10**

**Effects of Research Tasks on Authorship for Early and Late Respondents**

|  | Early 1 | Late 1 | Early 2 | Late 2 | Early 3 | Late 3 |
| --- | --- | --- | --- | --- | --- | --- |
|  | Author | Author | Author | Author | Author | Author |
| *Conception and Design, or Analysis and Interpretation of Data* | 2.825*** | 2.022*** |  |  |  |  |
|  | (0.234) | (0.232) |  |  |  |  |
| *Drafting and/or Revising the Article* | 2.442*** | 2.007*** |  |  |  |  |
|  | (0.217) | (0.245) |  |  |  |  |
| *Final Approval* | 3.438*** | 3.129*** |  |  |  |  |
|  | (0.247) | (0.267) |  |  |  |  |
| *Research Design* |  |  | 1.018*** | 0.840*** | 0.923*** | 0.763** |
|  |  |  | (0.221) | (0.238) | (0.222) | (0.240) |
| *Literature Search* |  |  | 0.455 | 0.693* | 0.381 | 0.629* |
|  |  |  | (0.259) | (0.285) | (0.259) | (0.287) |
| *Literature Analysis* |  |  | 0.622* | 0.659* | 0.609* | 0.642* |
|  |  |  | (0.271) | (0.303) | (0.271) | (0.305) |
| *Data Work* |  |  | 0.530** | -0.112 | 0.427* | -0.223 |
|  |  |  | (0.185) | (0.214) | (0.186) | (0.217) |
| *Results Description* |  |  | 1.169*** | 1.034*** | 1.120*** | 0.994*** |
|  |  |  | (0.240) | (0.271) | (0.240) | (0.274) |
| *Writing Paper* |  |  | 1.698*** | 1.340*** | 1.606*** | 1.276*** |
|  |  |  | (0.246) | (0.272) | (0.247) | (0.276) |
| *Remarking Paper* |  |  | 0.989*** | 0.376 | -0.408 | -1.059* |
|  |  |  | (0.211) | (0.240) | (0.390) | (0.457) |
| *Approving Paper* |  |  | 2.831*** | 2.713*** | 2.459*** | 2.307*** |
|  |  |  | (0.250) | (0.285) | (0.258) | (0.298) |
| *Remarking Paper and other research task(s)* |  |  |  |  | 1.909*** | 1.974*** |
|  |  |  |  |  | (0.443) | (0.519) |
| Chi2 | 447.21 | 325.77 | 450.67 | 327.36 | 451.39 | 329.16 |
| p > Chi2 | 0.000 | 0.000 | 0.000 | 0.000 | 0.000 | 0.000 |
| Observations | 3744 | 2387 | 3744 | 2387 | 3744 | 2387 |

* p < 0.05; ** p < 0.01 *** p < 0.001

Coefficients correspond to the marginal effects for the independent variables calculated at the mean levels of the remaining variables derived from mixed-effects logistic regressions with standard errors in parentheses.
